# Integrating whole-genome resequencing and machine learning to refine QTL analysis for fruit quality traits in peach

**DOI:** 10.1093/hr/uhaf087

**Published:** 2025-05-23

**Authors:** Jiaqi Fan, Jinlong Wu, Pere Arús, Yong Li, Ke Cao, Lirong Wang

**Affiliations:** National Key Laboratory for Germplasm Innovation & Utilization of Horticultural Crop, The Key Laboratory of Biology and Genetic Improvement of Horticultural Crops (Fruit Tree Breeding Technology), Zhengzhou Fruit Research Institute, Chinese Academy of Agricultural Sciences, Ministry of Agriculture and Rural Affairs, 500 meters south of the intersection of Hanghai Road and Weilai Road, Guancheng Hui District, Zhengzhou 450009, China; National Key Laboratory for Germplasm Innovation & Utilization of Horticultural Crop, The Key Laboratory of Biology and Genetic Improvement of Horticultural Crops (Fruit Tree Breeding Technology), Zhengzhou Fruit Research Institute, Chinese Academy of Agricultural Sciences, Ministry of Agriculture and Rural Affairs, 500 meters south of the intersection of Hanghai Road and Weilai Road, Guancheng Hui District, Zhengzhou 450009, China; Zhongyuan Research Center, Chinese Academy of Agricultural Sciences, No. 28 Hongqiqu Road, East Hall, Building 2, Chuangzhi Gongyuan, Henan Testing and Inspection Industry Park, Pingyuan Demonstration Zone, Xinxiang 453599, China; Institute of Agrifood Research and Technology (IRTA), Campus UAB, Edifici CRAG, Cerdanyola del Vallès (Bellaterra), 08193 Barcelona, Spain; Centre for Research in Agricultural Genomics (CRAG), CSIC-IRTA-UAB-UB, Campus UAB, Edifici CRAG, Cerdanyola del Vallès (Bellaterra), 08193 Barcelona, Spain; National Key Laboratory for Germplasm Innovation & Utilization of Horticultural Crop, The Key Laboratory of Biology and Genetic Improvement of Horticultural Crops (Fruit Tree Breeding Technology), Zhengzhou Fruit Research Institute, Chinese Academy of Agricultural Sciences, Ministry of Agriculture and Rural Affairs, 500 meters south of the intersection of Hanghai Road and Weilai Road, Guancheng Hui District, Zhengzhou 450009, China; National Key Laboratory for Germplasm Innovation & Utilization of Horticultural Crop, The Key Laboratory of Biology and Genetic Improvement of Horticultural Crops (Fruit Tree Breeding Technology), Zhengzhou Fruit Research Institute, Chinese Academy of Agricultural Sciences, Ministry of Agriculture and Rural Affairs, 500 meters south of the intersection of Hanghai Road and Weilai Road, Guancheng Hui District, Zhengzhou 450009, China; National Key Laboratory for Germplasm Innovation & Utilization of Horticultural Crop, The Key Laboratory of Biology and Genetic Improvement of Horticultural Crops (Fruit Tree Breeding Technology), Zhengzhou Fruit Research Institute, Chinese Academy of Agricultural Sciences, Ministry of Agriculture and Rural Affairs, 500 meters south of the intersection of Hanghai Road and Weilai Road, Guancheng Hui District, Zhengzhou 450009, China; Western Research Institute, Chinese Academy of Agricultural Sciences, No. 195 Ningbian East Road, Changji, Changji Hui Autonomous Prefecture, Changji 831100, China

## Abstract

Increasing marker density results in better map coverage and efficiency of genetic analysis. Here, we resequenced a large (*N* = 235) F1 progeny from two distant peach cultivars, ‘Zhongyou Pan #9’ and ‘September Free’, and constructed two parental maps (1:1 segregations) and one combined map (1:2:1 segregations) with 134 277 SNPs. Markers with the same genotype for all individuals studied were grouped in bins and a unique genotype for each bin was inferred to avoid mapping problems derived from erroneous data. The total genetic distance of the two parental maps was 431.9 and 594.2 cM with a short mean distance, 0.9 cM, between contiguous bins (groups of markers with the same genotype) and high collinearity with the peach genome. The genetics of eight fruit-related traits was analyzed for 2 years, allowing the positions of two major genes, fruit shape (*S*) and flesh adhesion to the stone (*F*), to be established, along with nine quantitative trait loci (QTLs) for quantitative traits including fruit soluble solids concentration, titratable acidity, weight, maturity date, and flesh color (yellow to orange). We developed a machine learning-based linear model to assess flesh color, which proved more efficient than physical colorimetric parameters (L, a^*^, b^*^), detecting consistent QTLs. Based on map position, gene expression patterns, and function, candidate genes were identified. Overall, our results provide two new elements: ultra-high-density maps with resequencing data to enhance mapping resolution and phenotyping strategies based on machine learning models that improve the quality of quantitative measurements to help understand the genetic control of key fruit quality traits.

## Introduction

Peach [*Prunus persica* (L.) Batsch] is one of the most economically valuable fruit trees globally. Due to its diploid compact genome (2n = *2x* = *16*), and its self-compatibility, peach is also a model organism to study drupe crops [1]. Research on peach genetics has experienced unprecedented growth since the completion of the peach genome sequence [2], generating a wealth of information on the inheritance of key traits that provided fundamental knowledge to help understand the origin and evolution of peach as a crop and provide tools for more effective plant breeding [3, 4].

Quantitative trait locus (QTL) analysis based on linkage maps and trait phenotypes obtained in biparental populations is one of the main strategies for dissecting and characterizing the inheritance of phenotypic variation. As technology advances, linkage maps are becoming more refined, allowing deeper exploration of trait genetics. The first peach genetic map was constructed with an F2 progeny using mainly random amplified polymorphic DNA (RAPD) markers [5], but these markers proved unreliable and nontransferable. Later, the first saturated peach genetic map was constructed using high-quality restriction fragment length polymorphism (RFLP) makers in an almond × peach F2 progeny [6]. This map proved to be a valuable reference for *Prunus* research, establishing standard terminology for chromosome number and orientation and providing high-quality markers, transferable to peach and other *Prunus* populations. Additional high-quality markers, such as simple-sequence repeats (SSRs), have been added to this map, allowing map comparisons across different rosaceous crops [7].

With a reference genome sequence available [2], the shift to single nucleotide polymorphisms (SNPs) and the use of SNP chips [8] provided researchers with improved tools to construct maps and to study population structure, evolution, and phylogenetic relationships [3]. With the continuous decline in sequencing costs, resequencing data, which provide a comprehensive view of DNA variation across the entire genome, are progressively becoming the primary tool in genomic analysis. Linkage maps based on whole-genome resequencing are being constructed in plant species, some of which are perennial crops, such as *Paulownia* and wolfberry [9, 10].

Using the combined information of biparental populations and genome-wide association studies (GWAS), >22 agronomic trait-related candidate genes or QTLs have been identified in peach [3, 4, 11, 12], most of them corresponding to major genes, known to segregate as Mendelian traits [13]. Many quantitative traits related to phenology and fruit quality are more challenging to study, but there have been clear advances in the elucidation of their component QTLs over the last decade [14, 15]. One of the limitations of analysis for complex fruit traits such as aroma and color is that they involve multiple interacting substances, requiring sophisticated quantitative methods as well as comprehensive statistical and bioinformatic analyses to be accurately assessed. For example, yellow fruit flesh is mainly due to accumulation of carotenoids [16], and while differences are noticeable, color cards and colorimeters are required for precise measurement. However, these methods can be subjective, are affected by light, and do not always agree with the L, a^*^, b^*^ color scale readings from colorimeters, indicating the need for more objective and reliable approaches for their assessment. Machine learning approaches are an opportunity to refine the phenotyping measurements, providing datasets with reduced environmental noise that result in a more accurate trait dissection and identification of causal genetic variants for complex agronomic traits [17, 18].

In this study, we provide results from two novel aspects that, individually or in combination, may increase the efficiency of QTL mapping in peach: the use of resequencing data to increase map resolution, and the integration of machine learning as a tool for more accurate phenotyping in one of the traits studied. We constructed a map with >130 000 SNPs segregating in the progeny of two distant peach cultivars using a mapping strategy that minimizes the complexity of dealing with an unusually high number of markers, with a concomitant increase in the number of erroneous datapoints. With this map, we analyzed data on eight agronomic traits and identified a set of QTLs that, in most cases, mapped to locations in agreement with previous studies. One of the traits, fruit flesh color from yellow to orange, studied for the first time in this paper, was characterized by a machine learning approach that identified two significant QTLs undetectable by conventional colorimetric methods. These QTLs were located at two genome regions identified as hotspots for peach fruit quality traits [14]. Biparental transcriptome data for genes in the locus interval were analyzed to screen for candidate genes controlling yellow to orange fruit color.

## Results

### Linkage map construction

**Figure 1:**
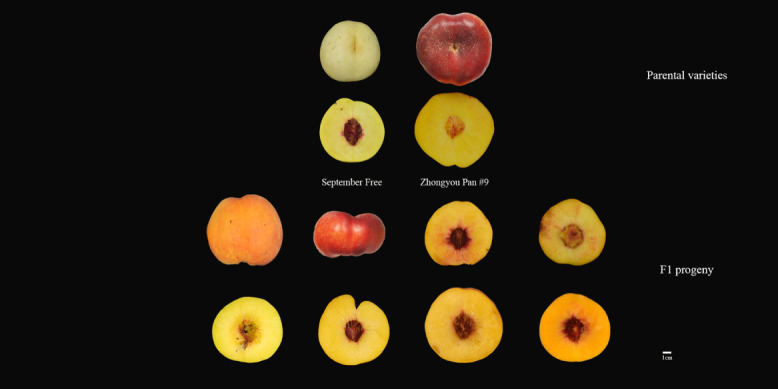
Fruit morphology of parents and F1 progeny of the ‘September Free’ × ‘Zhongyou Pan #9’ population.

Analyzing the whole-genome resequencing data of the parents and progeny of the ‘September Free’ × ‘Zhongyou Pan #9’ cross (SF × ZP) (Fig. 1), 1 002 519 high-quality SNPs were identified by aligning the sequences to the peach reference genome (v2.0.1). After marker filtering and linkage analysis, 134 277 segregating SNPs from the SF × ZP progeny were mapped to eight linkage groups (LGs) in each of the three maps constructed: the two parents (SF and ZP) and the combined map with 1:2:1 segregation (SF × ZP) (Table 1). We discarded three plants with an unexpectedly high number of recombination events, leaving a total of *N* = 232 individuals for further genetic analysis.

**Table 1:** Description of the linkage maps of ‘September Free’ (SF), ‘Zhongyou Pan#9’ (ZP), and the composite map including the 1:2:1 segregating markers (SF × ZP).

SF map						
Linkage group	nb. SNPs	nb. bins	Avg. SNPs /bin	% of Genome with IBD regions^a^	Genetic distance (cM)	Physical distance (%) covered
LG1	2898	62	47	41.4	65.8	99.3
LG2	9185	78	118	29.8	64.8	92.8
LG3	5010	54	93	12.4	54.7	98.1
LG4	13 284	71	187		49.9	95.9
LG5	596	30	20	62.6	39.2	37.4
LG6	7876	92	86		60.6	99.2
LG7	3443	59	58		61.8	99.5
LG8	6104	62	98		35.1	87.5
TOTAL	48 397	508	95	19.2	431.9	91.5
ZP map						
Linkage group	nb. SNPs	nb. bins	Avg. SNPs /bin	% of Genome with IBD regions^a^	Genetic distance (cM)	Physical distance (%) covered
LG1	13 466	138	98	54.8	158.1	99.8
LG2	1675	12	140	47.4	7.4	52.5
LG3	5808	86	68	29.9	81.7	98.7
LG4	7225	67	108	12.2	75.0	96.8
LG5	5251	71	74	35.1	42.8	64.2
LG6	8998	133	68	14.6	87.6	98.7
LG7	7046	70	101		78.5	99.9
LG8	9079	101	90		67.1	98.1
TOTAL	58 548	675	86	27.6	598.2	90.4
SF × ZP map						
Linkage group	nb. SNPs	nb. bins	Avg. SNPs /bin	Genetic distance (cM)		Physical distance (%) covered
LG1^b^	4392	106	41	77.3		75.2
LG2	4646	18	258	21.1		39.5
LG3^b^	435	29	15	10.2		11.2
LG4	6310	120	53	62.3		94.7
LG5	384	28	14	8.1		5.6
LG6	4546	99	46	74.0		96.1
LG7	5053	65	78	71.5		98.4
LG8	1564	67	23	47.3		73.7
TOTAL	27 330	489	51	371.8		64.5

a Percentage of the physical map of each chromosome covered with large gaps (>2.5 Mb) without polymorphic markers, i.e. regions possibly IBD.

b The linkage group was separated in two subgroups.

For the SF map, 48 397 polymorphic SNP markers were placed in 508 bins, with eight linkage groups spanning a total of 431.9 cM and an average interval of 0.9 cM/bin. These bins covered 91.5% of the physical genome, and 19.2% of the physical regions in the SF map were identified as possibly identical by descent (IBD). The ZP map contained 58 548 segregating SNPs distributed in 676 bins, with a total span of 598.2 cM and an average interval of 0.9 cM/bin. The genome coverage of the ZP map was 90.4%, and 27.6% of the genome was considered IBD. The SF × ZP map consisted of 27 332 SNPs, with a 1:2:1 segregation, divided into 489 bins with an average of 56 SNPs/bin. The total length of this map was 335.2 cM, with an average interval of 0.7 cM/bin and a genome coverage of 67.7% (Table 1). A high number of markers per bin were identified in the two parental maps (95 for SF and 86 for ZP), allowing for an accurate and usually complete determination of the genotype of each bin. The resolution of the map was also high: considering only the non-IBD regions, we estimated that the average physical size per bin was 0.29 Mb.

**Figure 2:**
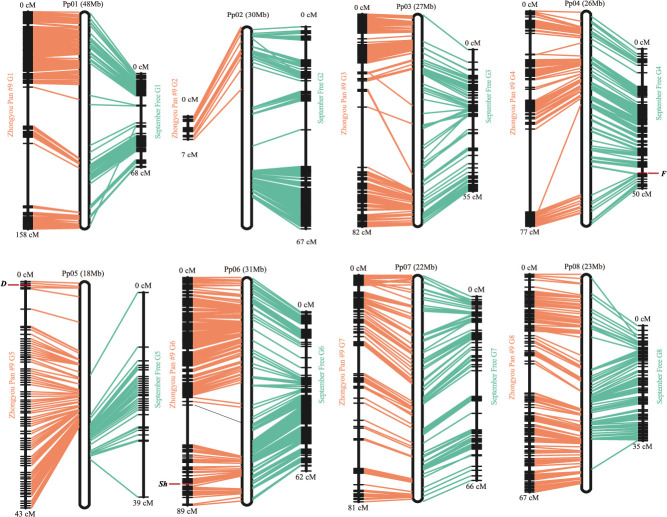
Linkage maps of the ‘September Free’ (SF, right-side connecting lines) and the ‘Zhongyou Pan #9’ (ZP, left-side connecting lines) parental lines, showing collinearity of the eight linkage groups with the dihaploid ‘Lovell’ v2.0.a1 peach reference genome (Pp01–Pp08).

There was high collinearity between the linkage maps and between the physical and linkage maps (Fig. 2). These maps also provided information useful for completing the peach reference genome v2.0.1: a total of 91 polymorphic SNPs enabled anchorage of 36 from the total of 182 unassembled scaffolds (19.8%), increasing the length of this genome by 512.9 kb. Twenty-two, 20, and 6 scaffolds were anchored on the ZP, SF, and SF × ZP maps, respectively (Table S1).

### Distribution, correlation, and heritability of traits

Apart from two qualitative traits, fruit shape, round versus flat (*S*), fruit flesh adhesion, and clingstone versus freestone (*F)*, all the traits were quantitative. The distributions of these characters and the values for the two parents of the SF × ZP cross were given in Fig. S1 and Table S2. In most cases the distribution significantly departed from normal (Table S3), with the exception of soluble solids concentration (SSC) and fresh fruit weight (FW). Parental values such as titratable acidity (TA) and FW were positioned at the extremes of the distribution, and, in the case of maturity date (MD), even exceeded these extremes. In contrast, the fruit color comprehensive score (FCS), colorimetric measurements (L, a^*^, b^*^), and SSC had intermediate values.

Correlations between traits were shown in Table S4 and Fig. S2, with significant correlations between years for all except the colorimeter traits (L, a^*^, b^*^). For intertrait correlations, flesh adhesion had a highly significant negative correlation with FCS, whereas the flat shape positively correlated with SSC and negatively correlated with FW.

The broad sense heritability was intermediate in TA, MD, and the L, a^*^, and b^*^ parameters (H^2^ = 0.47–0.54) and higher in SSC and FCS at 0.68 and 0.76, respectively (Table S3).

### QTL analysis

Using a permutation test (see methods) we established that the logarithm of odds (LOD) values for the SF, ZP, and SF × ZP maps, corresponding to a threshold with *P*-value ≤0.05, were 2.5, 2.7, and 3.3, respectively. Analyses of all traits studied yielded a total of 31 significant QTLs (Table S5) in addition to the data of the two major genes, *S* and *F*, which were analyzed both quantitatively and qualitatively. Table 2 is a summary of the nine most relevant QTLs: seven were consistently identified in 2021 and 2022. To these we added two QTLs for FW, which we considered of interest, although this trait was only analyzed in 1 year (2021). Table 2 lists the QTLs from the SF × ZP map only when both parents segregated for the QTL, and where QTLs segregated in only one parent, the one detected with the highest LOD either in the parental or the combined map was listed.

**Table 2:** Description of the relevant QTLs detected in the ‘September Free’ (SF) × ‘Zhongyou Pan #9’ (ZP) progeny (SF × ZP) for the traits analyzed.

Trait	QTL^a^	Year	Map	Group	cM	Peak bin(s)^b^	Interval (Mb)^c^	LOD	%EV^d^	a^e^	d^f^	d/a	Gene action^g^
Soluble solid concentration	**qSSC5**	21	ZP	LG5	9.4	Pp05_5837752	5.1	2.9	14.7	−1.6			
		22	ZP	LG5	9.4	Pp05_5837752	3.1	3.0	9.6	−1.4			
Titratable acidity	**qTA5**	21	ZP	LG5	0.3	Pp05_399680	3.1	6.7	20.1	0.2			
		22	ZP	LG5	0.8–1.3	Pp05_528954-Pp05_1233539	3.2	7.5	22.6	0.3			
Fruit weight	qFW6.1	22	SF × ZP	LG6	0.0	Pp06_420298	0.4	3.6	12.0	−20.9	15.6	−0.74	**PD**
	qFW6.2	22	ZP	LG6	80.9	Pp06_28593122	2.5	9.1	27.5	45.2			
Maturity date	**qMD4**	21	SF	LG4	25.5–25.9	Pp04_10391147-Pp04_10511096	4.9	2.6	8.4	2.7			
		22	SF	LG4	26.8–27.1	Pp04_10683173-Pp04_10685025	2.2	4.5	12.9	5.8			
	**qMD6.1**	21	SF × ZP	LG6	8.5–8.7	Pp06_3842155-Pp06_3917767	2.8	4.9	15.2	−2.5	0.8	−0.30	**PD**
		22	SF × ZP	LG6	7.8	Pp06_3527862	3.3	5.7	16.3	−4.5	0.03	−0.01	**A**
	**qMD6.2**	21	SF × ZP	LG6	35.6	Pp06_13575103	3.0	5.8	17.7	−2.5	1.4	−0.58	**PD**
		22	SF × ZP	LG6	34.7–36.2	Pp06_13387740-Pp06_14200392	3.0	5.1	14.6	−4.5	−1.2	0.27	**PD**
Fruit color	**qFC4**	21	SF	LG4	26.8–27.2	Pp04_10683173-Pp04_10685025	0.9	7.6	22.7	−1.3			
		22	SF	LG4	22.2–22.6	Pp04_9059939-Pp04_9073528	5.4	6.3	19.2	−1.1			
	**qFC5**	21	ZP	LG5	0.0–1.2	Pp05_130874-Pp05_1233539	3.2	4.3	13.6	1.0			
		22	ZP	LG5	0.8–1.2	Pp05_528954-Pp05_1233539	3.2	4.8	15.0	1.0			

a Consistent QTLs (found both years) in bold.

b Representative marker(s) of the bin(s) at the LOD peak for each QTL.

c Physical distance between markers at the boundary LOD-1 of the peak value.

d %EV = percentage of phenotypic variance explained.

e a = additivity (A–B)/2 for 1:2:1 segregation and (A–H) for 1:1 segregation, where A and B are the mean values of the two homozygous individuals and H of the heterozygotes.

f b = dominance [H–(A + B)/2].

g Gene action: |0.75| ≤ d/a ≤ |1.25| D (dominant); |0.25| ≤ d/a ≤ |0.75| PD (partly dominant); −0.25 ≤ d/a ≤ 0.25, A (additive).

### Mapping the fruit shape and flesh adhesion major genes

The F1 progeny had 85 individuals with flat fruits and 91 with round fruits, in agreement with a 1:1 segregation (χ^2^ = 0.20; n.s.). Our analysis identified a highly significant QTL on LG6 located at 80.5 cM, with the LOD peak at the bin position Pp06_26 836 010, explaining >91% of the phenotypic variance (Table S5). This QTL was found on the flat peach parent ‘Zhongyou Pan #9’ map, as expected, as flat peach corresponds to the heterozygous genotype of the *S* gene, and the round shape, as in ‘September Free’ is homozygous. Using qualitative data this trait was located on the map as a marker in the same bin on LG6.

Regarding fruit flesh adhesion (*F*), controlled by a single gene with the freestone allele being dominant, the segregation of 80 clingstone:60 freestone did not significantly deviate from the expected 1:1 ratio (χ^2^ = 2.86; n.s.). When analyzed as quantitative, a distinct peak was identified on LG4 on the SF map at position 48.3 cM with a high LOD score and explaining 76% of the phenotypic variance, corresponding to the bin Pp04_18598676. *F* was located in the same bin when mapped as a single Mendelian character.

### QTL analysis of SSC, TA, and FW

**Figure 3:**
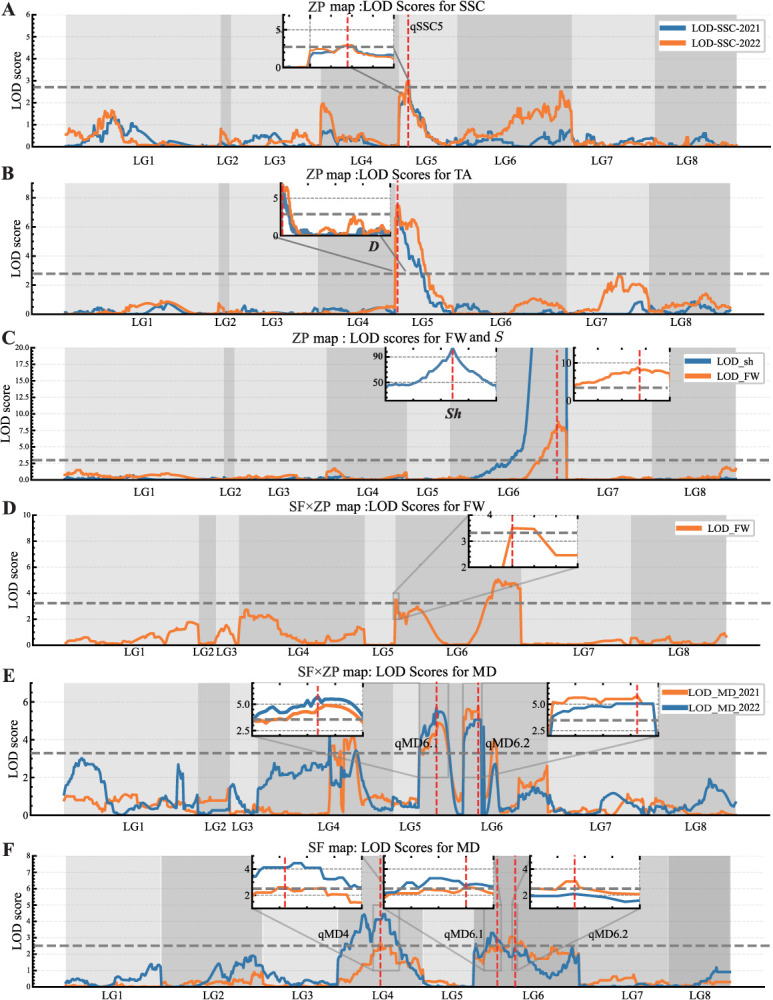
Genome-wide QTL analysis for various traits studied in 2021 and 2022 in the maps of ‘September Free’ (SF), Zhongyou Pan#9 (ZP), and SF × ZP. (A) Soluble solids concentration (SSC), (B) titratable acidity (TA), (C) fresh weight (FW) and shape (*S*), (D) FW, and (E, F) maturity date (MD). The gray dashed lines indicate the LOD threshold at *P* ≤ 0.05, which is 2.7 for ZP, 2.5 for SF, and 3.3 for SF × ZP.

A QTL for SSC on LG5, qSSC5, with the LOD peak in bin Pp05_5837752, was identified on the ZP map in both 2021 and 2022, with %EV = 9.6–14.7 (Table 2, Fig. 3A). Another QTL in a more proximal position of the same linkage group was detected for TA, qTA5, at 0.3 cM on the ZP map. In both years, this QTL explained 20.1%–22.6% of the phenotypic variance (Table 2, Fig. 3B), with peak LOD values corresponding to the physical positions between 0.4 and 1.3 Mbp on chromosome 5, overlapping with the region where the major gene *D*, which determines the acid versus subacid trait, is located.

Fruit weight was only studied in 2021, with two QTLs detected (Table 2, Fig. 3C and D). The QTL with the highest LOD score (9.1), qFW6.2, was located on the ZP map at 80.9 cM on LG6 (bin position Pp06_28593122), explaining 27.5% of the phenotypic variance. This QTL is mapped at the region of the *S* gene, which determines fruit shape (Fig. 3C). A second QTL, qFW6.1, was identified in SF × ZP near the proximal end of LG6, with the peak at 0.0 cM, corresponding to bin Pp06_420298 (Fig. 3D), with LOD = 3.6 and accounting for 12.0% of the phenotypic variance.

### QTL analysis of MD

The MD ranged from early August to late September over the 2 years. In 2022, progeny maturation was 7–10 days later than in 2021 (Fig. S1). Three consistent QTLs were identified: one on LG4 and two on LG6 (Table 2, Fig. 3E and F). The qMD4 QTL was significant in the SF and SF × ZP maps, while the two QTLs on LG6, qMD6.1 and qMD6.2, had the highest LOD scores in the SF × ZP map and were significant in both the SF and ZP maps (Table S5). For qMD4, the QTL peak (LOD = 2.6 in 2021 and 4.5 in 2022) was located between bins Pp04_10391147 and Pp04_10686025 (25.5–27.1 cM), explaining 8.4%–12.9% of the phenotypic variance. The second QTL, qMD6.1, was positioned near the proximal end of LG6, spanning from Pp06_3527862 to Pp06_3917767, with LOD scores ranging from 4.9 to 5.7 and accounting for 15.2%–16.3% of the phenotypic variance. The LOD peak of the third QTL, qMD6.2, was 5.1–5.8, located in the central region of chromosome 6 between bins Pp06_13387740 and Pp06_14200392 (34.7–36.3 cM), explaining a similar percentage of phenotypic variance (14.6%–17.7%) as qMD6.1. The gene action for these two QTLs was additive to partly dominant (Table 2). Each allele of these three QTLs produced a phenotypic effect on maturity dates ranging from 2.5 to 4.5 days, and their combined effects resulted in changes of between 7.4 and 12.1 days when considering only additive effects (Table 2).

### Fruit color evaluation and QTL mapping with machine learning models

**Figure 4:**
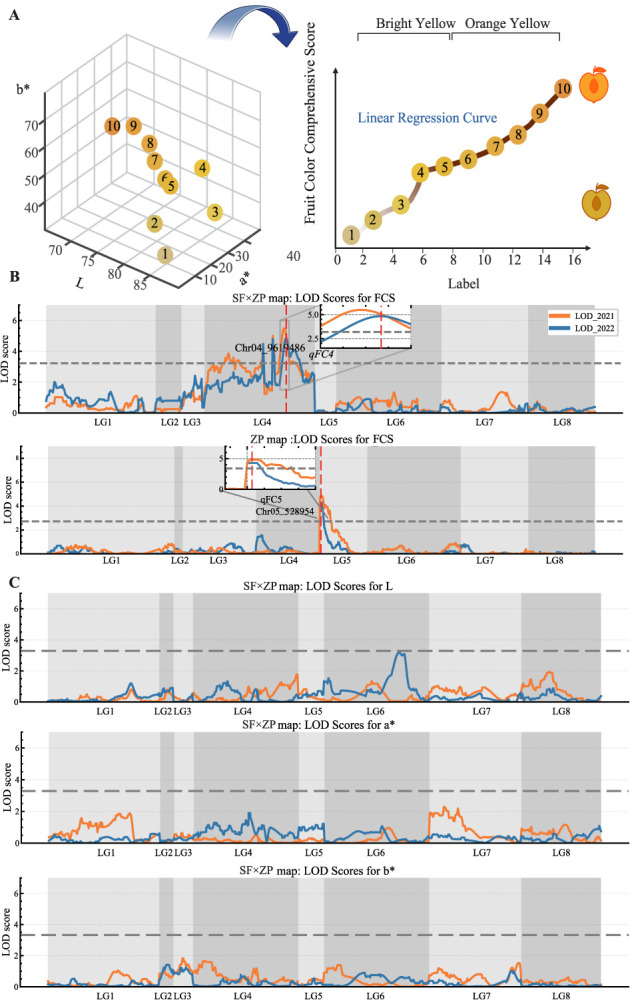
Genome-wide QTL mapping of peach yellow to orange flesh color intensity in the maps of ‘September Free’ (SF), Zhongyou Pan#9 (ZP), and SF × ZP. A) 3D representation of 10 gradations of yellow to orange in peach flesh (left panel), illustrating the range and intensity of color variation, with a linear regression model predicting yellowness shown in the right panel. B) FCS, where FCS represents the quantified intensity of peach flesh color based on the machine learning model. C) QTL mapping using three physical color parameters—L (lightness), a^*^ (red/green axis), and b^*^ (yellow/blue axis). The gray dashed lines indicate the LOD threshold at *P* ≤ 0.05, which is 2.7 for ZP, 2.5 for SF, and 3.3 for SF × ZP.

The parents and progeny of SF × ZP were phenotyped for the physical color parameters L, a^*^, and b^*^ and for the machine learning-derived FCS (Fig. 4A). The ripe fruit of ‘Zhongyou Pan #9’ was orange-yellow (FCS = 5.8), while the ripe fruit of ‘September Free’ was light yellow (FCS = 3.4) and the progeny had a range of FCS between 1.5 and 7.5 (Fig. S1). To evaluate the robustness of FCS, a 5-fold cross-validation was conducted, resulting in an average coefficient of determination (*R^2^*) of 0.969 (±0.01), mean absolute error (MAE) of 0.211 (±0.02), and root mean squared error (RMSE) of 0.267 (±0.03).

 Data for L, a^*^, and b^*^ were used for QTL mapping in both 2021 and 2022, but no consistent QTLs were identified (Fig. 4C). In contrast, analysis based on FCS scores revealed two consistent QTLs, qFC4 and qFC5. The qFC4 QTL was located in the central region of LG4 (22.2–27.2 cM), corresponding to bins Pp04_9059939 to Pp04_10685025, on the SF map for both years, and accounted for ~20% of the phenotypic variation (Table 2, Fig. 4B). High LOD scores (6.3–7.6) were found in the SF map, whereas in the ZP map there was no significance, indicating that ‘September Free’ was heterozygous for this QTL, while ‘Zhongyou Pan #9’ was homozygous. The other QTL, qFC5, was detected at 0.0–1.2 cM, with the LOD peak at the physical interval of bins Pp05_130874 to Pp05_1233539. The LOD scores ranged from 4.3 to 4.8, explaining 13.6%–15.0% of the phenotypic variance. This QTL was identified in the ZP map but not in the SF map, suggesting that only ‘Zhongyou Pan #9’ was heterozygous.

Based on the QTL confidence intervals for fruit coloration (FC) (Table 2), a total of 970 genes were identified (693 in qFC4 and 277 in qFC5), with 189 differentially expressed genes (DEGs) between the two parents (out of 268 genes listed in Table S6). To narrow down potential candidate genes, we compared the functional annotations of these genes and incorporated transcriptome data from the parents during fruit development across five stages of fruit color change resulting in a total of 189 selected candidates (Table 6S). Candidate genes related to the synthesis and regulation of carotenoids and anthocyanins and transcription factors were further explored and identified as potential candidates in the confidence intervals of qFC4 and qFC5, including one abscisic acid 8′ hydrolase (*Prupe.5G013100*) and 10 transcription factors members of the bZIP family (*Prupe.5G003000*, *Prupe.4G172000, Prupe.5G027000*), the ERF family (*Prupe.4G222300, Prupe.4G176200*), the NAC family (*Prupe.4G187100, Prupe.4G186800*), the MYB family (*Prupe.4G215500, Prupe.4G192000*), and the WRKY family (*Prupe.4G217900*) (Table S6).

## Discussion

### Resequencing data allowed the construction of ultra-high-density linkage maps

Linkage map resolution in biparental populations depends essentially on two factors: i) the total number of recombination events detectable, which increases with the size of the population and the degree of heterozygosity of the parents, and ii) the density of markers, with higher densities making it possible to establish the positions of these recombination events with more precision. Population sizes rarely exceed *N* = 100–200 in peach and other perennial species, due in part to their slow growth and large plant sizes that often imply a high cost per individual. Marker chips with thousands of SNPs have become available in peach [8, 19] and the construction of saturated linkage maps with the same markers in different populations is now less challenging [20]. Increasing marker density is still an affordable option with the use of SNPs detected using resequencing data from the individuals of the mapping population. In this paper, we provide the densest map constructed to date in a fruit tree species with >130 000 polymorphic SNPs obtained from resequencing data of a large F1 segregating progeny.

Handling such a large amount of data has several problems, one being the need for adequate mapping software tools [21], specifically when dealing with F1 segregating populations. However, the most important challenge is to accurately deal with the large amount of erroneous and missing data, which we solved using an approach based on the bin concept [22], with which we have obtained robust maps of reasonable lengths and highly syntenic with the peach genome sequence, with some gaps that we attributed to IBD regions that are common in peach cultivars due to the high co-ancestry of elite materials [23–25]. These gaps may affect entire or almost entire chromosomes as occurs in chromosome 2 of the ZP and in chromosome 5 of the SF map. The map constructed with 1:2:1 segregations covered a smaller part of the peach genome compared to the parental maps, which was expected considering that 1:2:1 segregations occurred when both parents were heterozygous, and therefore could not cover the IBD regions of either or both parental maps. In this study, SNP markers were grouped into bins, requiring various time-consuming steps that were done manually. Automation of the process, specifically adapted to large datasets, is a desirable development, and we are currently working on the production of a user-friendly, robust platform for large-marker datasets (>10 000 markers per chromosome).

### Positions of major genes and QTLs supported the existence of hotspots for peach fruit quality traits

Given the high resolution of our map (on average 239–353 kb/bin when using each parental map and 144 considering both parental maps together), the positions of the major genes can be precisely identified. The fruit shape *S* gene is located in a 1.72-Mb bin of Pp06_26 836 010, ranging from 26 836 010 to 28 557 142 bp, with the flanking bins separated by 1.3 cM. This fragment contains three candidate genes, namely *Prupe.6G292200, Prupe.6G290900*, and *Prupe.6G323700*. We could discard a fourth candidate gene, *Prupe.6G262200* [26], as it was located in the upstream bin of our map, one recombination from the bin containing *S*, in agreement with previous results [27]. Most of the span of the Pp06_26 836 010 bin (97%) included the position of a 1.67-Mbp inversion associated with the flat versus round trait [28]. The lack of recombination in this region in ZP confirms the presence of heterozygosity in the inverted segment as predicted considering its flat fruit. Eleven recombination events were identified in the same region of the SF map, considering that ‘September Free’ is homozygous and recombination is not suppressed in this region.

The *F* gene was detected in an unusually large bin (Pp04_18598676) of 4.30 Mbp (3.1 cM between flanking bins) in LG4 of the SF map. The bin contains the candidate gene for this trait (*Prupe.4G262200*), coding for an endopolygalacturonase [29]. The bin containing the *F* gene had 6510 SNPs with roughly even coverage of all its physical distance (maximum gap 0.21 Mbp) and was >14 times longer than the average physical size of the bin in this map (0.29 Mbp), suggesting that this region has a low recombination frequency in SF. When comparing the homologous region of the ZP map, we found that only the distal region of 0.72 Mb (1.6 cM) was segregating and the rest was part of an IBD fragment. The segregating region was covered by five bins equivalent to an average distance of 0.14 Mbp per bin, which was about twice more recombination than the average of these maps, suggesting a dramatic decrease of recombination frequency in the heterozygous genotype of ‘September Free’, compatible with the presence of one or several inverted DNA fragments.

Other traits known to be determined by major genes were analyzed as quantitative, such as the acid versus subacid gene, *D*, measured here as TA, and known to be located at the beginning of chromosome 5, with candidate genes estimated to be within the first megabase of this chromosome [11, 30, 31]. The QTL peaks identified were in this region on the ZP map (Table 2), as expected considering that this was the parent with low acidity, since the subacid phenotype is provided by a dominant allele. Similarly, the maturity date *MD* gene, at the center of chromosome 4, is within the interval of qMD4 (Table 2) and has a strong candidate gene: an NAC transcription factor (*Prupe.4G186800*) involved in the ethylene biosynthesis pathway located at Pp04:11116814–11 118 655 [32, 33].

QTLs identified for other traits were in regions compatible with the positions of these four major genes. This is the case of qFC4 and qFC5, colocating with *MD* and *D*, respectively. The regions encompassing these two genes are known to be hotspots of genes of agronomic interest in the peach genome [3, 14, 15], containing QTLs for many important traits of peach fruit phenology and quality. These include fruit development period, fruit weight, redness around the pit, flesh adhesion, ripening date, fruit firmness, and SSC on chromosome 4 (within an interval estimated by da Silva Linge et al [14] to be 9.0–12.5 Mb), and fruit texture, pH, SSC, and TA on chromosome 5 (0.3- to 3.7-Mb interval). In some cases, there is clear evidence for the existence of separate candidates for different QTLs in these hotspots, as occurs with the fruit acidity (*D*) locus, with two candidates *Prupe.5G004300* [11] and *Prupe.5G006300* [30] and the *DBF* (dominant blood flesh) locus [34] determining red flesh color (*PpBL* or *Prupe.5G006200*) [32]. This may be the case for the SSC consistent QTL that we found on LG5 (qSSC5), at a different position to the *D* locus, but within the boundary of the chromosome 5 hotspot [14].

A QTL of particular interest for plant breeding is one for fruit weight (qFW6.1) located at the proximal end of chromosome 6. This is a dominant QTL, and the allele of interest generated an additive gain of 20 g per fruit in the SF × ZP progeny. The same QTL has been repeatedly found in peach × peach and peach × almond progenies [15, 27, 35, 36] and deserves more detailed analysis to understand its molecular basis. Another fruit weight QTL (qFW6.2) was mapped at the end of chromosome 6, the position of the *S* gene. These two traits were highly correlated, with roundness strongly associated with heavy fruits (Fig. S2), suggesting that the most likely reason for their comapping is pleiotropy.

Maturity date is a quantitative trait with usually high heritability [14] and determined by various QTLs. As previously mentioned, one these, qMD4, coincides with the position of the *MD* gene a NAC transcription factor is a strong candidate (Prupe.4G186800), which has an allele with major effects that explains qualitative changes in maturity date [32, 33] and is associated with a 9-bp in-frame insertion [33]. Interestingly, the parents of SF × ZP were fixed for the presence (ZP) and absence (SF) of this 9-bp insertion, and all the progenies were heterozygous. In addition, the qMD4 was detected only in the SF map. These observations suggest that other alleles at this gene or other genes in the neighborhood with minor effects on this trait were responsible for the variability determined by qMD4. This also explains why the maturity dates of both parents were extreme and above the distribution of variability observed in their progeny (Fig. S1). Two more consistent QTLs, qMD6.1 and qMD6.2, were found on LG6. Only qMD6.2 has been detected previously [37], while we believe that the other, qMD6.1, is described here for the first time.

### Machine learning facilitates inheritance analyses of complex traits such as peach flesh color

Machine learning has demonstrated its strength in stably quantifying continuous variables and reducing subjectivity in phenotypic surveys [38]. In this study, we used a typical graded assessment of continuous variation, analogous to methods used in plant stress studies [39]. We found that directly using a single dimension of color space (L, a^*^, b^*^) was ineffective, failing to identify meaningful QTLs. We hypothesize that this is because L, a^*^, and b^*^ are overly sensitive since they are used to capture the entire spectrum of visible colors [40, 41]. Visual assessment is also problematic, as it is prone to subjective errors [42, 43], which can significantly impact the consistency of quantitative trait measurements and their reproducibility across different years [17].

Supervised learning, a type of machine learning [44], involves the model learning the relationship between input features (L, a^*^, b^*^ values in this study) and known outputs (FCS in this study) to make predictions [45]. The simple linear regression model produced stable results with a small training set [46, 47] and was effective in fruit color grading, producing stable QTLs. There are several notable limitations. First, the sample size of ~300 fruits may still be insufficient to capture the full genetic and environmental variability of yellow peach coloration, potentially constraining the model’s robustness. Second, by focusing on linear combinations of L, a^*^, and b^*^, the model may overlook more complex or nonlinear interactions. Exploring advanced approaches such as random forests or neural networks could reveal additional insights. Additionally, while our lab-based color measurements are standardized, they may not fully align with on-site conditions or consumer perception. Minor variations in lighting or measurement protocols could introduce discrepancies not accounted for here. Lastly, our FCS system was tailored to yellow peaches and might require recalibration for other cultivars or fruit types. Nonetheless, these initial findings demonstrate the feasibility of using a straightforward regression model for color-based QTL mapping, providing a foundation for more extensive and diverse follow-up studies [48, 49].

The QTLs responsible for anthocyanic fruit skin and flesh color have been repeatedly localized to the positions where we found qFC4 and qFC5 [12, 14, 15, 35, 50, 51]. A strong interaction between a gene determining peach blood flesh (*PpBL*), located within the interval of qFC5, and a major gene for maturity date (*MD*) colocating with qMD4 was reported [32, 52], where blood-fleshed individuals generally had earlier maturity dates than white-fleshed. We examined our progeny to see if a similar pattern occurred with the yellow to orange variation. We categorized MD data over 2 years into early and late groups, following the approximately bimodal distribution of this trait (see Fig. S1), and considered as late those individuals with MD = 243 days or later in 2021 and MD = 249 days or later in 2022 with the rest being early-ripening. We found that early-ripening fruit had, on average, a deeper color: in 2021, FCS = 4.7 for early fruit and FCS = 4.3 for late fruit, and in 2022, FCS = 4.3 and 3.6 for early and late fruit, respectively (Fig. S4). In both cases, the early group was significantly darker than the late group (one-tailed *t*-test; 2021: *P* < 0.05; 2022: *P* < 0.01). These results suggest a parallelism between the inheritance of red-to-white and orange-to-yellow flesh color in peaches. Interestingly, *PpBL (Prupe.5G006200)*, considered a key transcription factor in blood peach [32], had minimal expression during fruit development in both parents of the SF × ZP population (Table S6). In contrast, the differential expression of *MD (Prupe.4G186800)*, an NAC transcription factor involved in peach fruit maturity date [33] and the slow-melting (nonripening) phenotype [24], was significant in the parents of SF × ZP at all developmental stages studied. Specifically, *MD* had maximum expression in ‘Zhongyou Pan #9’ during the first stage of fruit development examined, whereas in ‘September Free’, this occurred in the fourth stage (Fig. S3). However, the lack of segregation of the major *MD*-related variant (a 9-bp insertion at *Prupe.4G186800*) in SF × ZP suggests that MD is heterozygous in all individuals of this progeny. Then, *PpBL* and *MD* are unlikely candidates to explain yellow to orange fruit color variation in SF × ZP, and other factors at these two hotspots of fruit quality genes may determine its inheritance. Qualitative analysis of these two plant pigments is required for more insight into the molecular basis of this character to determine whether only carotenoids are involved in yellow versus orange determination or it is influenced by both carotenoid and anthocyanin accumulation.

Our results on expression of genes located at qFCS4 and qFCS5 regions may be a source of candidate genes: e.g. *Prupe.5G013100*, encoding an abscisic acid 8′-hydroxylase known to be involved in regulating downstream pathways of β-carotenoid metabolism in *Arabidopsis* [53], may enhance the carotenoid degradation pathway. This gene was downregulated during fruit development in both parents. Other examples are the four NAC and ERF transcription factor family members identified, which are involved in ethylene response, and had significant differential expression during the fruit development of both parents, or *Prupe.4G215500* of the MYB family with substantial differences in expression of the parents during the different ripening stages (Fig. S3).

## Conclusions

Here we constructed a robust and ultra-high-density linkage map with >130 000 SNPs obtained from whole-genome resequencing data of progeny from two phenotypically and genetically distant peach cultivars. The strategy was based on the redundant information provided by contiguous markers to facilitate error-free genotyping, prior to introducing the data in the mapping software. This allowed location of qualitative and quantitative trait loci responsible for the inheritance of a set of agronomic traits with precision. Many of the positions of QTLs and major genes confirmed the inheritance data of previous work, validating the adequacy of the mapping strategy used. Other QTLs were in new positions and allowed a better understanding of the genetics of the traits analyzed. In addition, a method based on machine learning was implemented to phenotype color intensity, a character difficult to measure, specifically different grades of yellow to orange color in fruit flesh. With this machine learning approach two QTLs, undetectable using conventional colorimetric measurements, were identified. We have shown that increased map resolution and enhanced phenotyping accuracy are two critical elements for understanding the genetics of complex traits in peach. Similar developments in other crop species are also likely to produce useful information that can rapidly be translated into more efficient breeding strategies.

## Materials and methods

### Plant materials

A mapping population from the cross between ‘September Free’ (female parent) and ‘Zhongyou Pan #9’ (male parent), SF × ZP, was generated, in 2017, at the Experimental Station of Zhengzhou Fruit Research Institute, Chinese Academy of Agricultural Sciences, Zhengzhou, Xinxiang, China. The phenotype of the two parents differs substantially (Fig. 1): ‘Zhongyou Pan #9’ is a sweet, flat clingstone peach with light yellow skin at maturity when bagged, dark red when unbagged, and orange-yellow flesh. It ripens at the beginning of July. ‘September Free’ is a freestone peach with high acidity, green skin at maturity when bagged, creamy white skin when unbagged, and bright yellow flesh. It ripens in mid-September. There was marked segregation of these traits in the hybrid progeny, making it suitable for genetic studies.

A total of 305 seedlings were grown under uniform management. We selected 235 F1 individuals (fruit-producing and with valid pedigrees) to construct the genetic linkage map for QTL mapping. Mature fruits harvested from each plant were used to measure various fruit traits. Fruit maturity was determined based on external and internal indicators of whether the fruit on the tree had reached physiological maturity: checking the skin color, aroma, flesh flavor, and fruit firmness. The management and sampling timing followed methods from our previous studies [54], with the main difference being that only mature fruits were sampled in this study.

### DNA extraction, resequencing, and SNP identification

Resequencing of the genome of 235 progenies and their parents was performed using genomic DNA extracted from young leaves, at an average depth of ~20x, as previously described [55]. At least 5 Gb of sequencing data were generated for each individual with the Novaseq 6000 platform. Raw reads were aligned, sorted, deduplicated, and subjected to variant calling against the reference genome following published procedures [12]. The filtered SNPs were reformatted to retain only segregating markers in the progeny.

### Genetic map construction

A hard-filtered SNP genotyping file was carefully curated to ensure sequencing depth and quality. SNPs were named first with Pp (for *P. persica*), then the chromosome number (01–08), followed by a dash and the physical position of the SNP in the dihaploid ‘Lovell’ genome v2.0 (e.g. Pp06_13387856) [56]. The SNP genotyping file, aligned with the reference genome, contained a substantial number of monomorphic markers in the SF × ZP population. To focus exclusively on polymorphic markers, we first retained only biallelic sites (i.e. SNPs with one reference allele and one variant allele) using the `bcftools view -m2 -M2` command in software bcftools (v 1.18) [57]. Following this, monomorphic markers were excluded, including SNPs where the parent genotype was missing or both parents were homozygous. Offspring genotypes that were inconsistent with parental genotypes were considered as missing data, and SNPs were excluded if the proportion of missing genotypes among the offspring exceeded 20%. Marker data were then converted into AHB terminology (where A and B are homozygous genotypes for the alternative alleles and H is heterozygous). Polymorphic markers were further filtered based on the following criteria: markers exhibiting a single genotype in the progeny were discarded, as were markers with an A genotype frequency f(A) > 0.9 or f(A) < 0.1, or with an H genotype frequency f(H) < 0.15 to ensure that the remaining segregating markers did not exhibit severe segregation distortion.

LepMap3 software [58] was then used to establish linkage groups with the filtered markers, with the LOD score threshold set to ≥19 in the ‘SeparateChromosomes2’ module, and segregating markers were categorized into eight linkage groups. For each linkage group, only markers known to be located on their chromosomes were retained, while the remaining markers were placed in a separate file for further analysis. Three datasets were prepared for each linkage group using the pseudo-test cross strategy [59], two for the parental maps (termed SF for ‘September Free’ and ZP for ‘Zhongyou Pan #9’), expected to segregate 1:1 and based on the genotypes of each parent, and the third dataset with all markers that were heterozygous in both parents that were expected to segregate 1:2:1 (the SF × ZP map). The two 1:1 segregating marker datasets were used to create the parental maps (SF and ZP), while the 1:2:1 dataset was used to create the combined map.

Given the large sizes of these datasets, the main problem was the many putative erroneous datapoints that would detect spurious double recombination (DR) events in short physical distances of the same chromosome. To minimize this problem, we used a multistep process based on establishing groups of markers with the same genotype, i.e. ‘bins’, using the terminology of Vision et al. [22], separated from the next bin by one or more recombination events. The process involved i) sorting SNPs by their physical order, ii) discarding markers with obvious genotype divergence with the rest of the SNPs in their position, or repositioning when possible, iii) visual phasing of the two 1:1 datasets (phasing was not applied to the SF × ZP dataset), and iv) identification of recombination breakpoints and separation of bins. These bins had a limited level of internal variability that we assumed was due to genotyping errors: a DR was accepted only if it was detected by at least two contiguous markers, and the physical distance of the fragment between the first and the last marker involved in the DR was ≥100 kb. A new file (the ‘one bin’ file) was constructed with only one marker per bin, where the first SNP (the one closest to the top of the chromosome) of each bin was used as the representative for each bin. Missing or contradictory datapoints of this marker were imputed with the most frequent genotype of the rest of the bin. For bins with a single marker, genotypes detecting a DR were considered missing data. The ‘one bin’ file was used for map construction using JoinMap 5 software [60] with the BC1 mode for the SF and ZP maps and the CP (cross-pollinating population) mode for the SF × ZP data. The Kosambi distance function and regression mapping algorithm were used, keeping other parameters at default settings.

The proportion of physical genome coverage was calculated as the distance between the first and last mapped markers on each chromosome, expressed as a percentage of the total chromosome length from the di haploid ‘Lovell’ reference genome [57]. Regions without polymorphic markers spanning ≥2.5 Mb (~1% of the genome) in the SF and ZP maps were considered possibly IBD. The IBD proportion was calculated for each chromosome and the entire genome. Collinearity analyses and visualizations were using ALLMAPS software [61].

### Quantitative trait locus and major gene mapping analysis

QTL analysis was performed with MapQTL (v6.0) software [62] using the interval mapping method. QTLs were retained when they had a significant threshold with probability *P* ≤ 0.05. The permutation test of MapQTL was used to calculate what was the LOD score corresponding to this threshold for each of the three maps with 10 000 permutations. It was expected that if a QTL was significant in both parental maps, it would also be significant in the SF × ZP map, indicating that both parents were heterozygous for the QTL. In this scenario, the SF × ZP map data would likely yield the highest LOD score and explain the largest percentage of variance (%EV). Alternatively, if a significant LOD score was found in the SF × ZP map and that of only one parent, this suggested that the QTL was segregating in just one parent. In such cases, the LOD and %EV values would be similar between the two maps or higher in the parent’s map. When a QTL was identified in the same region on multiple maps, such as SF and SF × ZP or ZP and SF × ZP, we reported the data from the map with the highest LOD score. We did not consider as relevant QTLs that were significant only in the SF × ZP map. The confidence interval for each QTL was determined using the flanking markers at the position of the maximum LOD, reduced by one LOD unit. If the confidence interval extended into an IBD region, we considered the beginning of that region as the endpoint for the confidence interval. Gene action was estimated by calculating the ratio between the ‘d’ (dominant) and ‘a’ (additive) values from MapQTL, following previously established criteria [63].

Major genes were analyzed as quantitative with the MapQTL software. When we found very sharp and high LOD scores, data were treated as single loci and mapped as any other marker.

### Trait phenotyping

Five to eight fruits at the same maturity stage were collected from the periphery of the fruit trees in 2021 and 2022. Fruit shape (flat vs round) and flesh adhesion (clingstone vs freestone) were analyzed after the maturity date following the evaluation criteria reported by Wang et al [52]. For SSC, FW, and TA, we adhered to the methods already described [11, 64]. The MD was the number of Julian days at harvest. For fruit color assessment, the fruit was cut vertically at the midpoint of the suture. The L, a^*^, and b^*^ values were directly measured using a handheld colorimeter (Linshang LS171, China), with at least three replicates per measurement and annual data recorded as the average. In addition, a color chart with 10 yellow shades—from light to dark—was used to grade the fruit. In the initial stages, the yellow grade was determined by directly comparing the fruit flesh with the chart.

Correlation tests and visualizations for all traits in this study were done using OriginPro (Version 2022, OriginLab, Northampton, Ma, USA). Frequency distribution histograms were constructed for the phenotypic data of all traits measured using the ‘matplotlib’ library in Python. The normality of the data was assessed using the Shapiro–Wilk test in Python using the ‘scipy.stats’ module.

### Development of a color grading evaluation system for yellow peaches using machine learning models

To establish a consistent grading standard for the color depth of yellow peaches, we developed a machine learning model using linear regression. This model was trained on color difference values derived from a standard color card representing 10 levels of yellow peach color. Specifically, >300 yellow peaches were sampled and their L, a^*^, and b^*^ values were recorded, corresponding to these 10 predefined grades. The collected dataset was then split into 70% for training and 30% for testing. While most samples fell within the 10 predefined grades, several additional test samples extended beyond these grades to evaluate the FCS.

To further mitigate overfitting and assess model stability, a 5-fold cross-validation was performed on the training set. In this procedure, the training set was randomly divided into five folds; for each iteration, four folds were used for model training and the remaining fold was reserved for validation. After five iterations, each fold served exactly once as a validation set, enabling us to calculate the mean and standard deviation of *R^2^*, MAE, and RMSE across all folds. Details on these metrics are provided in the reproducible script.

The model was implemented using the ‘LinearRegression’ function from the scikit-learn library in Python [65]. We defined the FCS prediction as:


$$ FCS={b}_0+{b}_1L+{b}_2a+{b}_3b $$

where ${b}_0$, ${b}_1$, ${b}_2$, and ${b}_3$ are the coefficients of the model, representing the intercept and the coefficients for each input feature (L, a^*^, b^*^), respectively. After confirming stable performance in the cross-validation phase, the final model was retrained on the entire 70% training subset. Its predictive accuracy was subsequently evaluated on the 30% held-out test set, using *R^2^*, MAE, and RMSE as primary performance metrics.

To ensure reproducibility, the Python scripts are provided in the Supplementary Material (‘fruit color evaluation.py’), and the training dataset is available in Table S7. These scripts allow users to input any [L, a^*^, b^*^] triplet—representing the color coordinates of a new sample—and obtain a corresponding FCS via the ‘model.predict()’ interface. This approach enables consistent comparison of color depth across different fruit samples. In our evaluation, the predicted FCS demonstrated a strong correlation with the visually assessed depth of yellow color, indicating the efficacy of our proposed grading system.

### RNA-seq library construction, gene expression analysis, and profiling

Samples were collected at five different stages of parental fruit development: 45, 65, 75, 85, and 95 days after anthesis, to obtain RNA-seq data. RNA was extracted, reverse transcribed, and expression levels of selected genes were quantified using quantitative polymerase chain reaction (qPCR) following the procedure used previously [66]. The cDNA libraries were constructed with the NEB Next UltraTM RNA Library Prep Kit (New England Biolabs, MA, USA). Pairwise RNA sequencing was performed using the Illumina HiSeq 2500 system, and the resulting transcriptome data analyzed with reference to the methodology [67].

### Gene selection and expression pattern analysis

All genes within the confidence intervals of qFC4 and qFC5 were selected for further analysis (Table S6). To identify potential candidate genes, transcriptome data from five developmental stages of the parental fruit were examined. Genes with a combined Fragments Per Kilobase of transcript per Million mapped reads (FPKM) value of ≤10 across all stages in both parents were filtered out, as they were considered to have minimal expression. The remaining genes were prioritized based on their functional annotations, with a focus on structural genes related to pigment pathways and transcription factors.

Further screening of transcription factors was carried out based on expression trends and statistical significance. We prioritized those with marked differences in expression patterns between the parents during the five developmental stages, particularly focusing on factors that were either consistently upregulated or downregulated. Additionally, transcription factors with similar expression trends but with significant differences at specific stages were also considered. Independent *t*-tests were conducted using the ‘ttest_ind’ function from the ‘scipy.stats’ module in Python, comparing the experimental group (‘Zhongyou Pan #9’) and the control group (‘September Free’) at each developmental stage. Significant differences in gene expression means were identified with a *P*-value threshold of <0.05.

## Supplementary Material

Web_Material_uhaf087

## Data Availability

The data that support the results are partly available in the paper and the supplementary materials published online. Resequencing data for the parents and the 235 progenies, markers used for mapping ordered in bins (the ‘bin’ set), and markers used for mapping with only one marker per bin (the ‘one bin’ set) for the ‘September Free’, ‘Zhongyou Pan #9’, and the SF × ZP (1:2:1) combined map are available in https://doi.org/10.6084/m9.figshare.27309288.v1. Sequencing data are in the Sequence Read Archive of the National Center for Biotechnology Information (NCBI) under BioProjects PRJNA1167467.
