# Supplementary figures and images for: Integrating whole-genome resequencing and machine learning to refine QTL analysis for fruit quality traits in peach
Source: Hortic Res. 2025 May 23;12(7):uhaf087. doi: 10.1093/hr/uhaf087 (PMC12365598; doi:10.1093/hr/uhaf087)

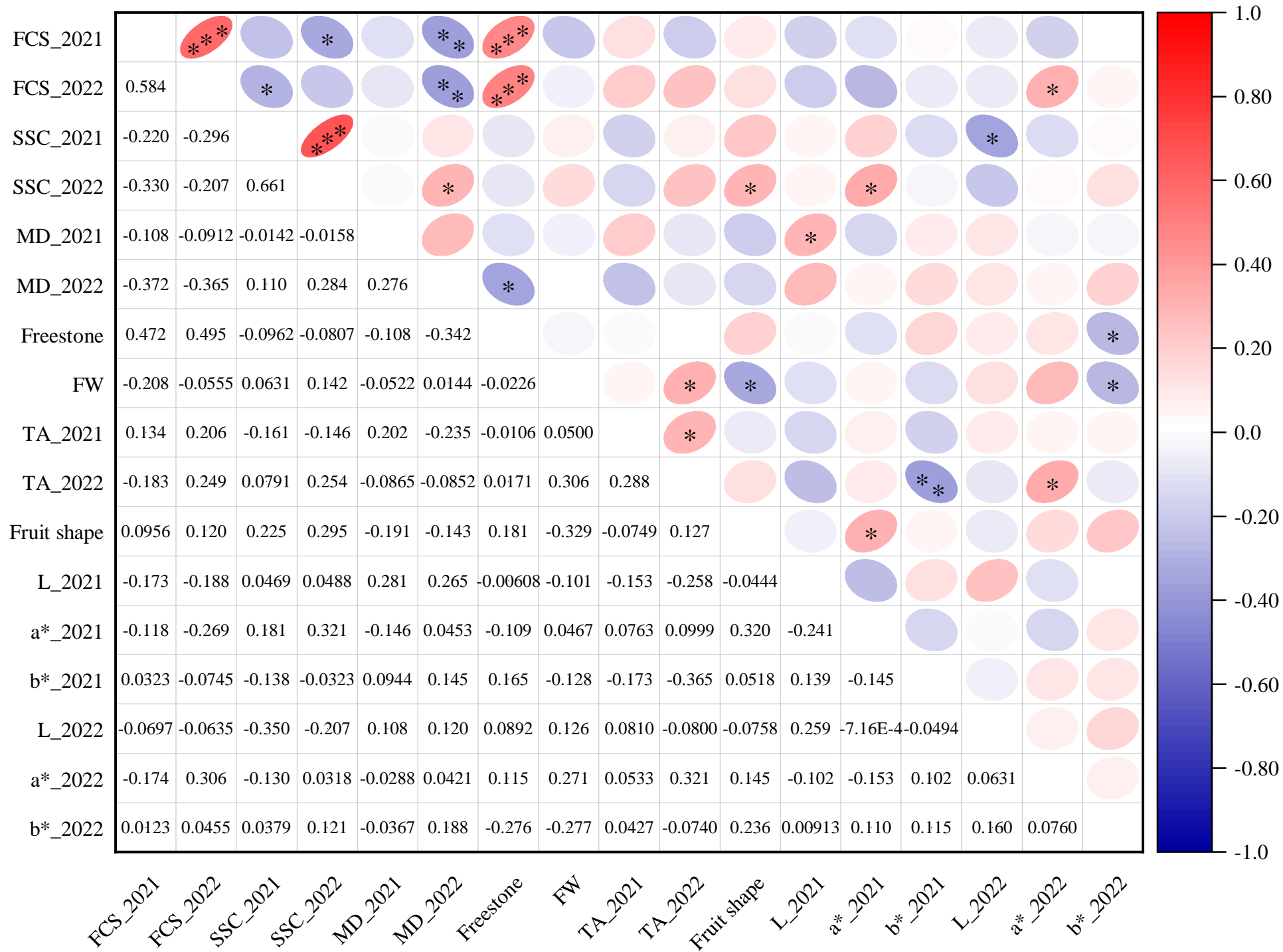

\* p<=0. 05

Supplement: Web_Material_uhaf087 [file Web_Material_uhaf087.zip › Fig.S2.pdf]

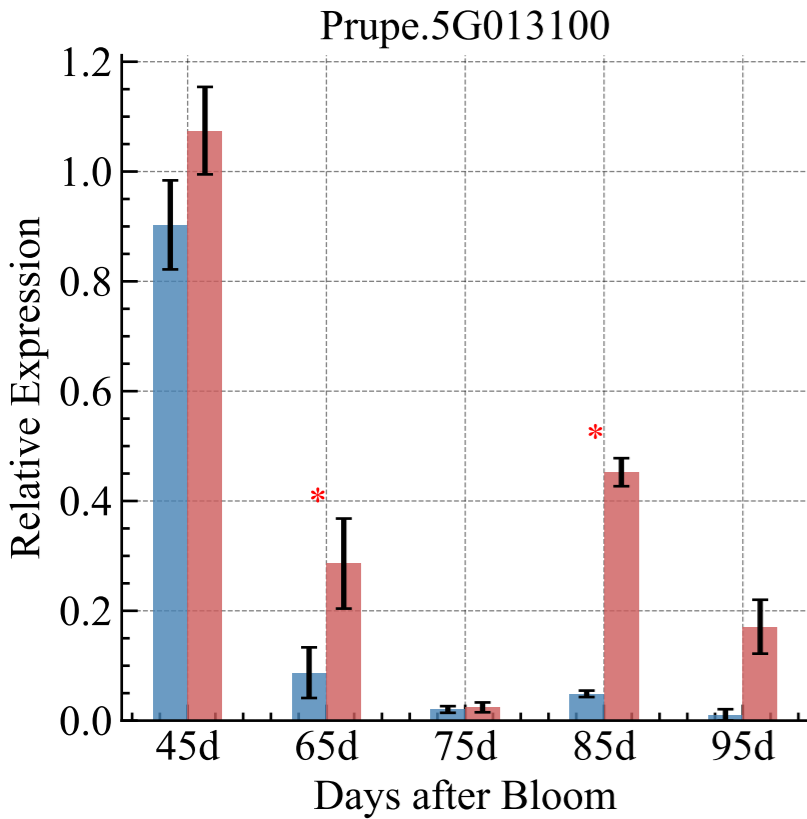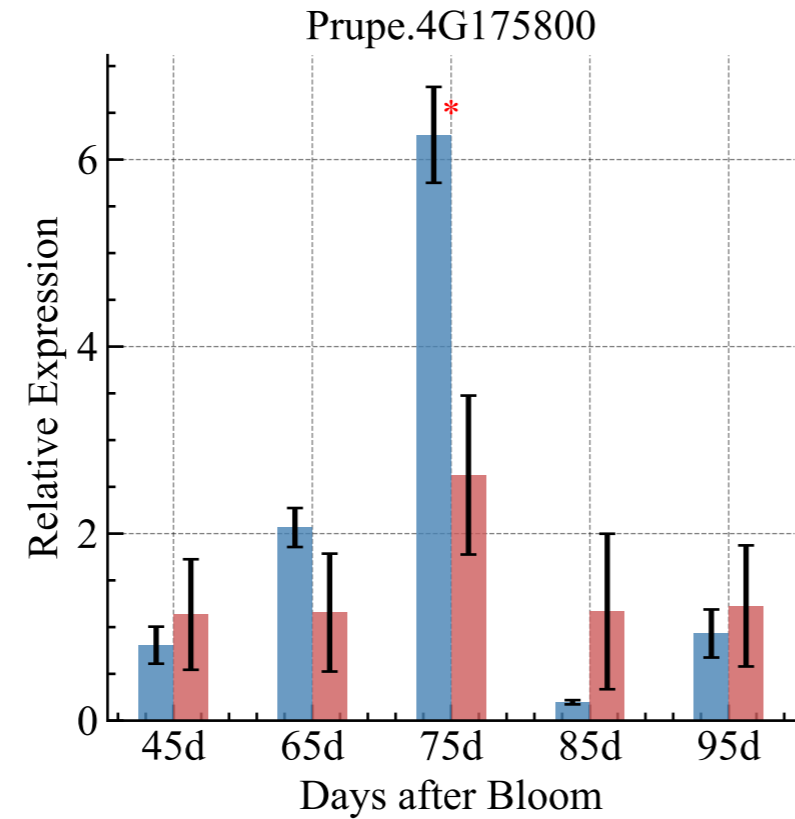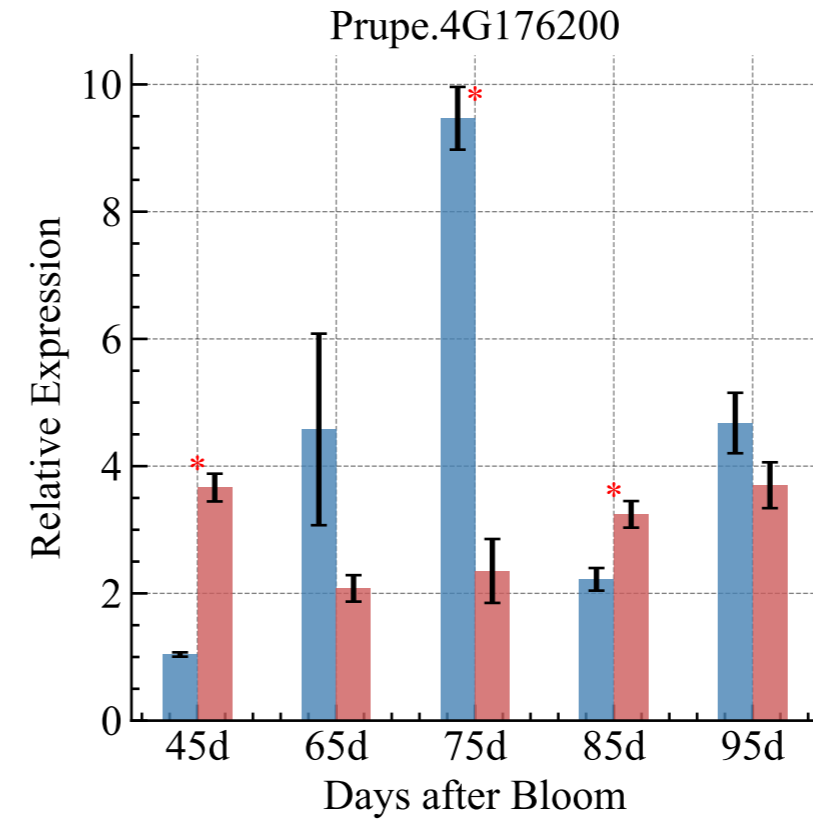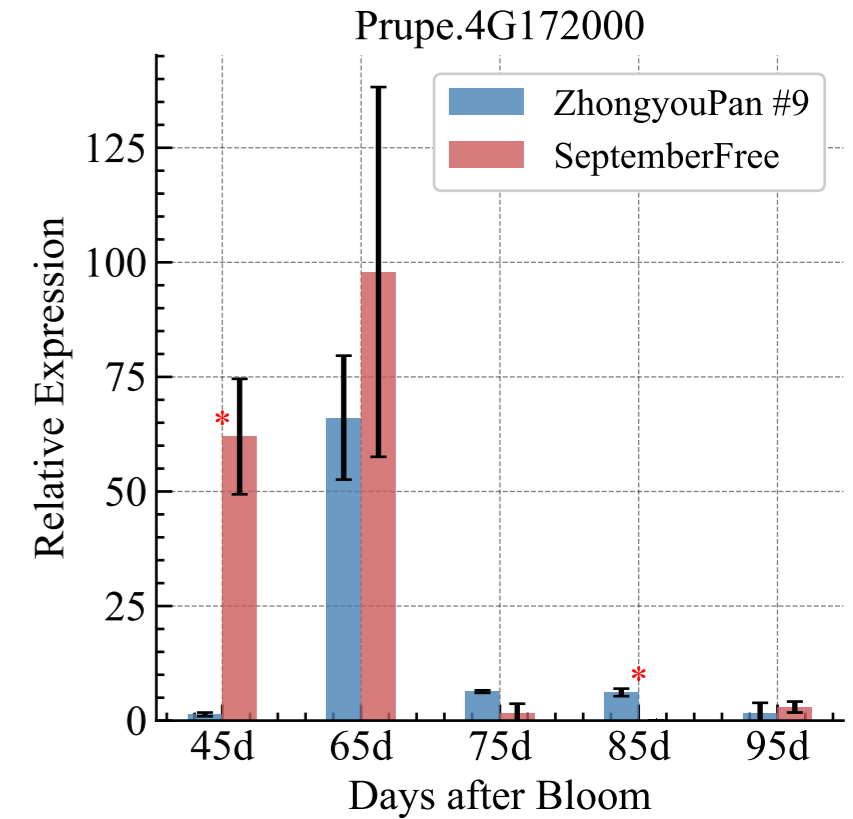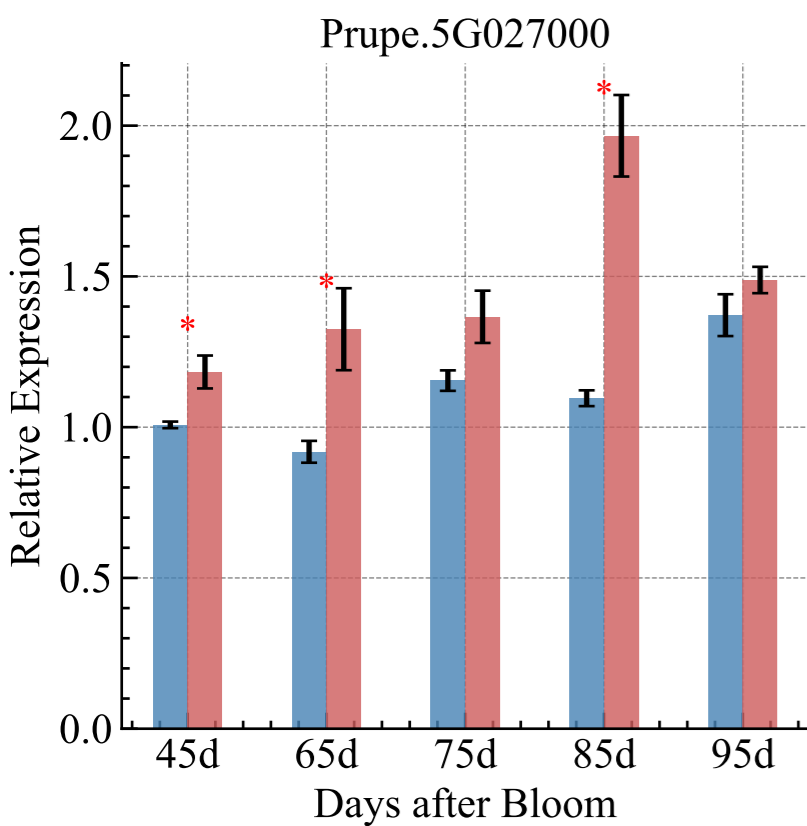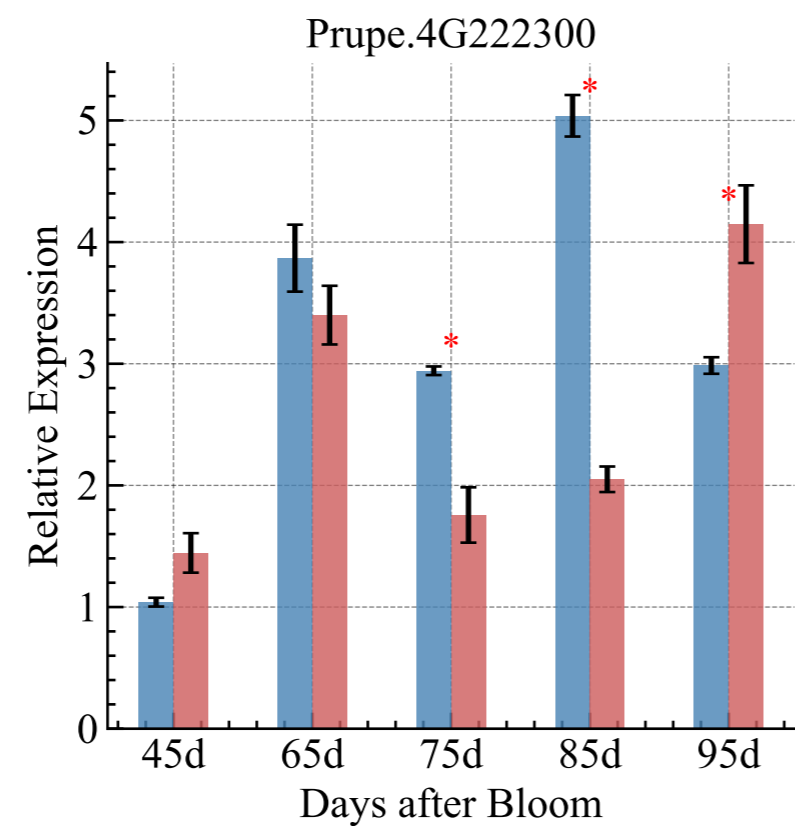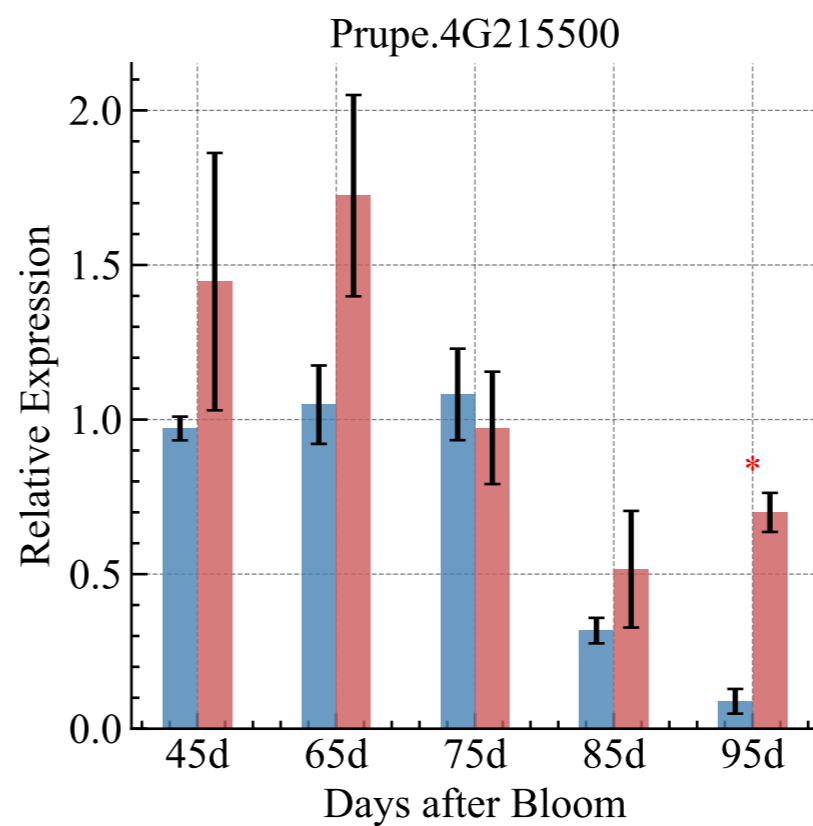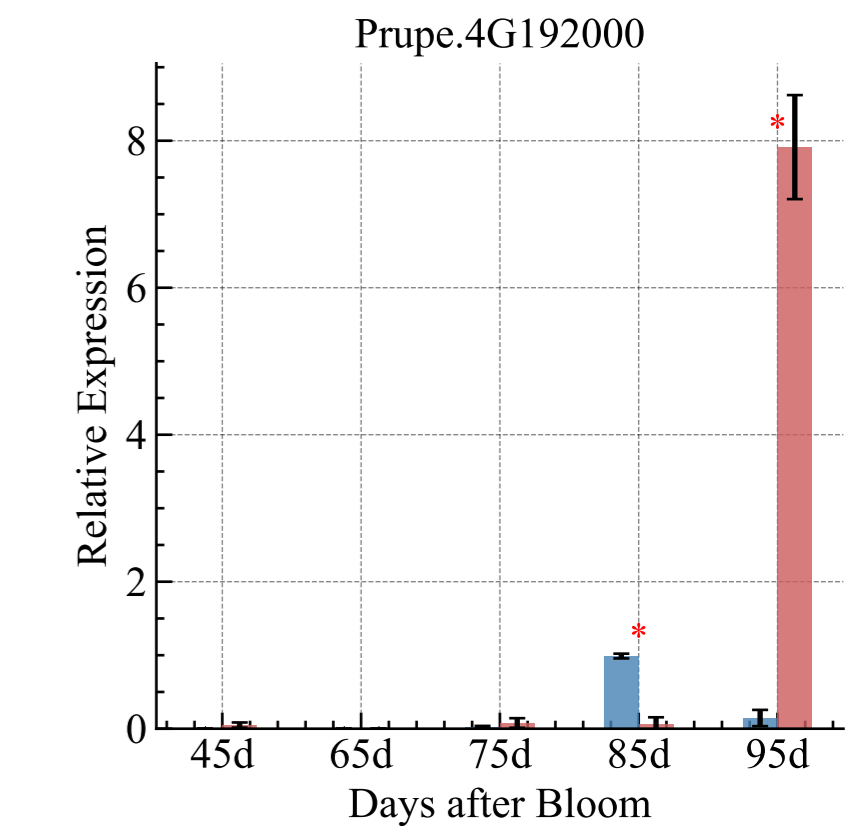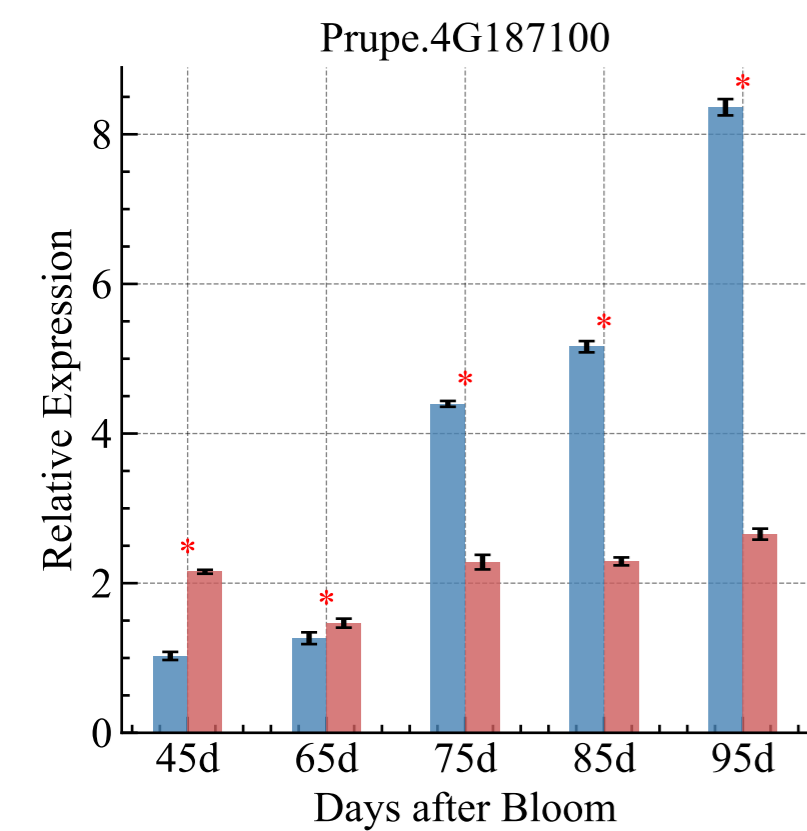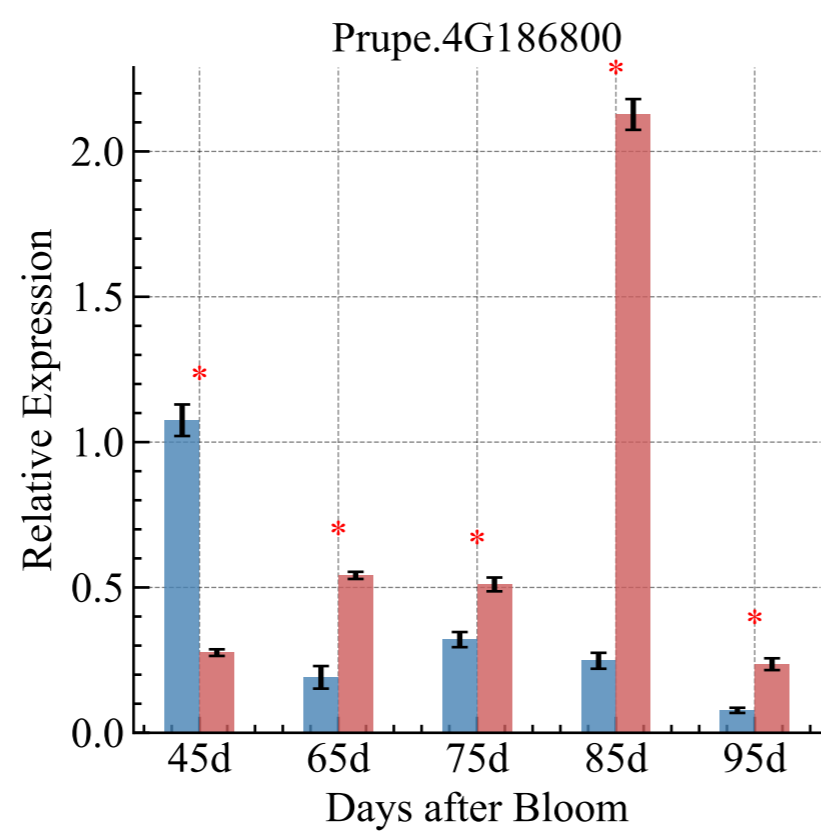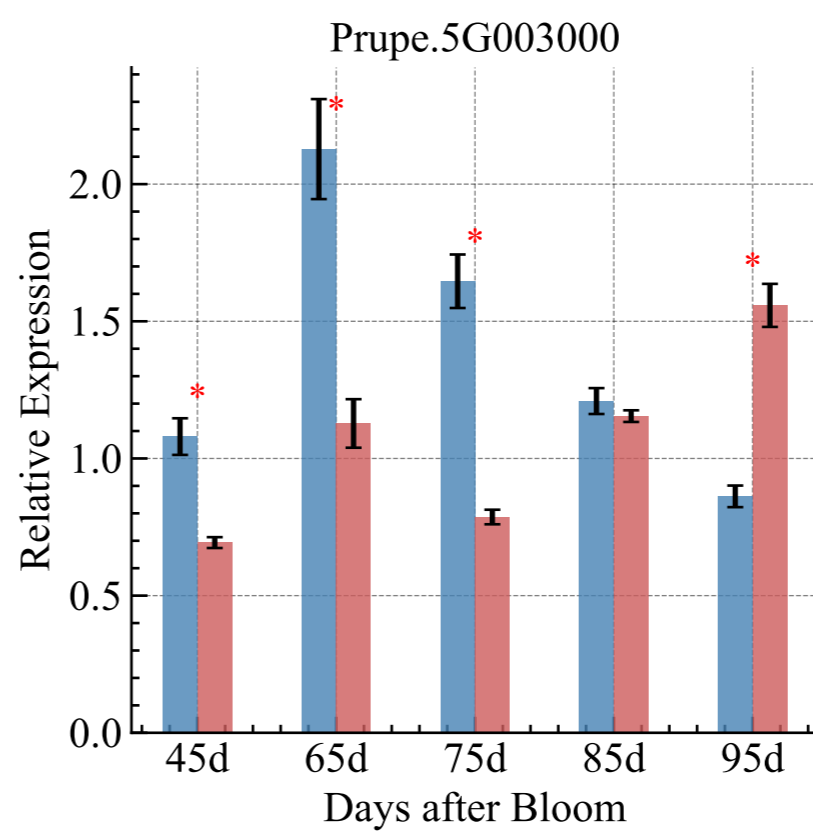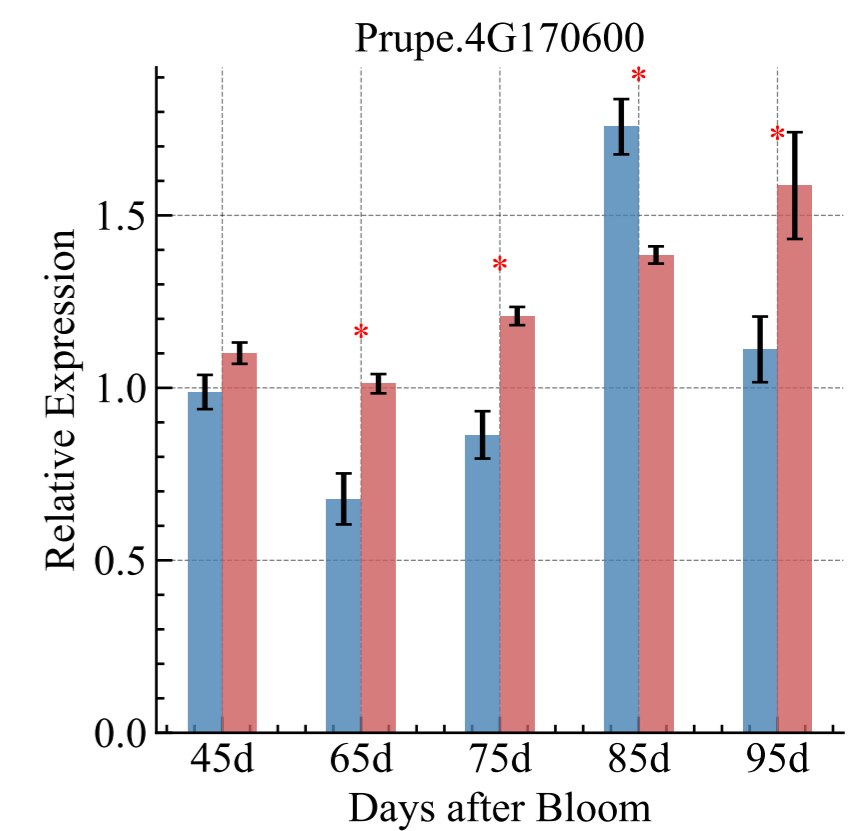

Supplement: Web_Material_uhaf087 [file Web_Material_uhaf087.zip › Fig.S3.pdf]

Boxplot of FCS across Maturity Stages (Late vs Early)

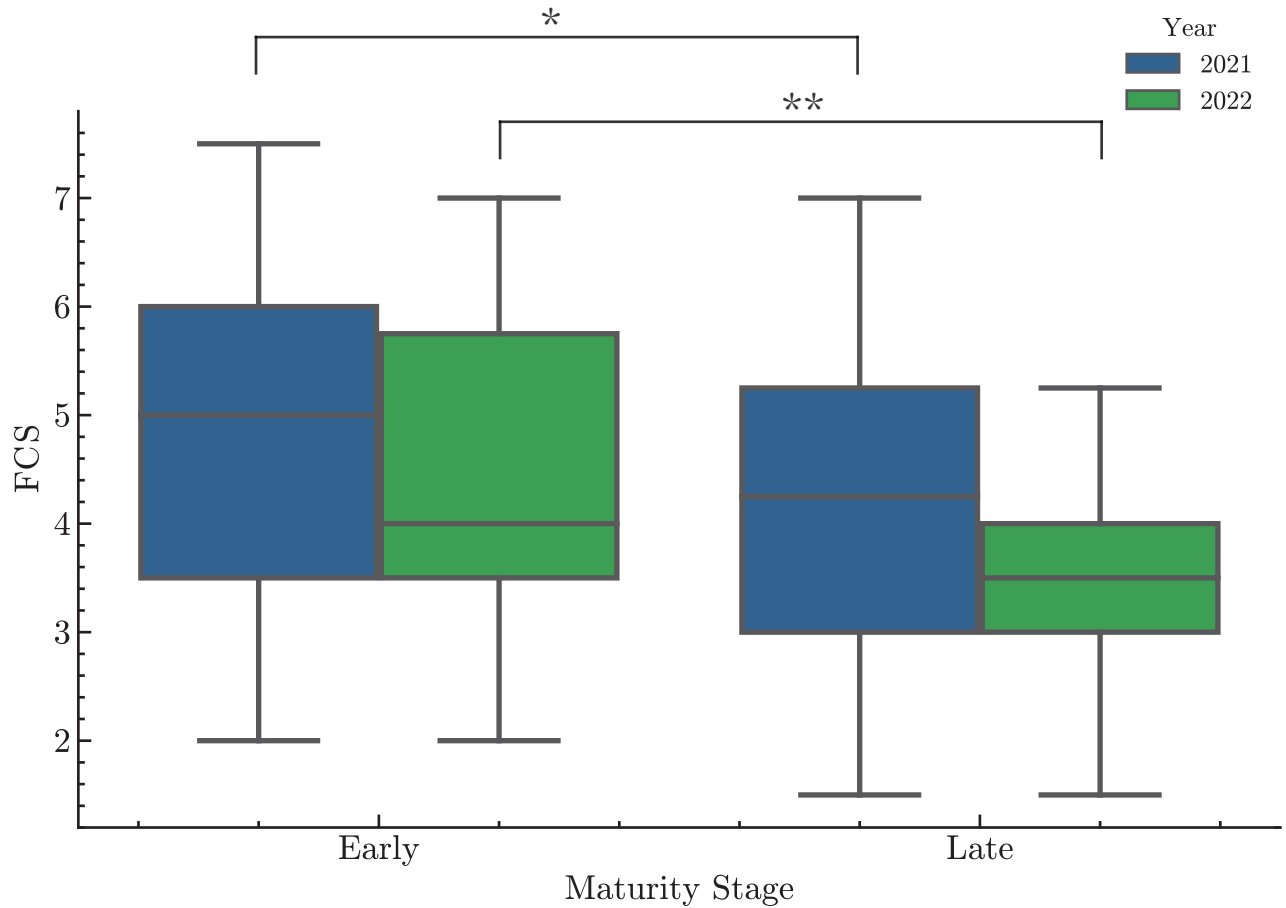

Supplement: Web_Material_uhaf087 [file Web_Material_uhaf087.zip › Fig.S4.pdf]

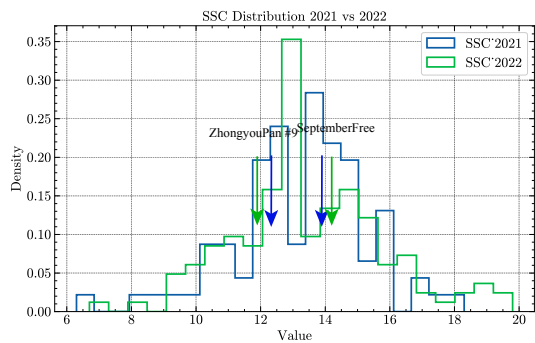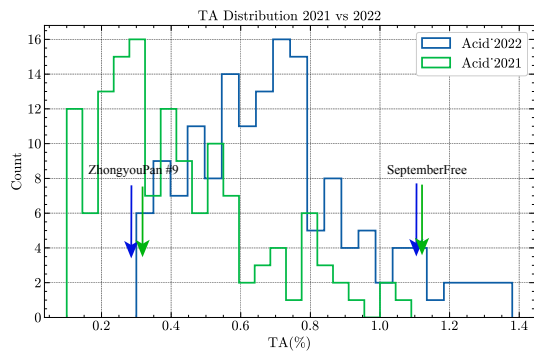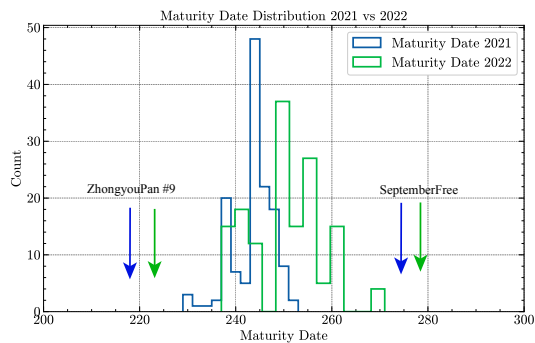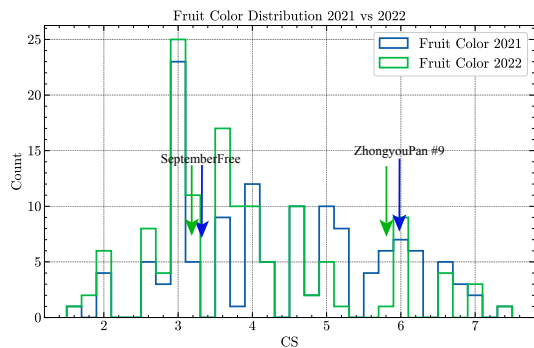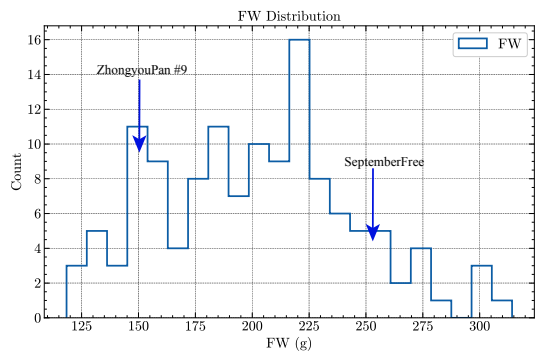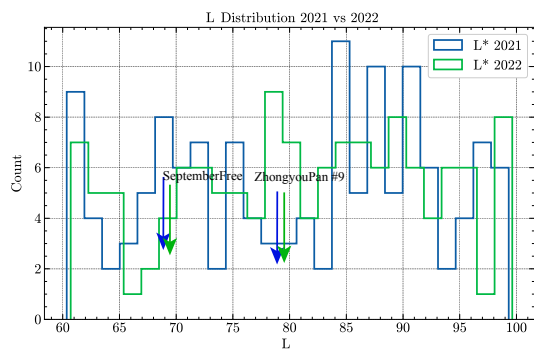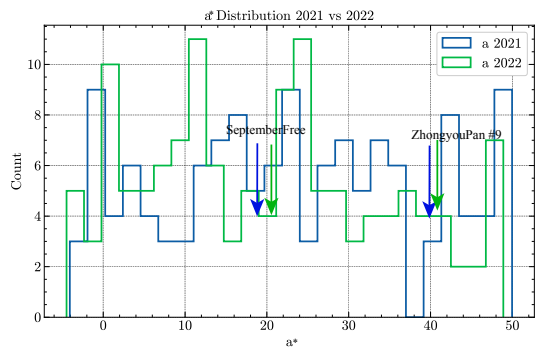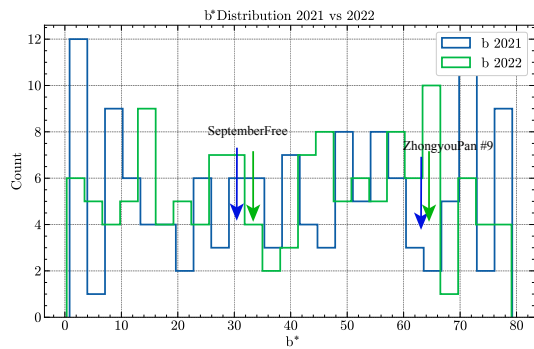

Supplement: Web_Material_uhaf087 [file Web_Material_uhaf087.zip › FigS1.pdf]
